# Change of Specimen Temperature during the Monotonic Tensile Test and Correlation between the Yield Strength and Thermoelasto-Plastic Limit Stress on the Example of Aluminum Alloys

**DOI:** 10.3390/ma14010013

**Published:** 2020-12-22

**Authors:** Adam Lipski

**Affiliations:** Laboratory for Research on Materials and Structures, Faculty of Mechanical Design, UTP University of Science and Technology, Al. prof. S. Kaliskiego 7, PL 85-796 Bydgoszcz, Poland; adam.lipski@utp.edu.pl; Tel.: +48-52-340-82-20

**Keywords:** monotonic tensile test, yield strength, thermoelasto-plastic limit stress, IR thermography

## Abstract

This paper presents an attempt to generalize the description of the course of specimen temperature changes during the tensile test and to connect the value of the thermoelasto-plastic limit stress with the value of a clear (physical) or proof strength (offset yield strength) on the example of tests of the following aluminum alloy sheets used in Poland for airplane structures: 2024-T3 and D16 in three grades: D16ATV, D16CzATV, and D16UTV. A thermographic camera was used for specimen surface temperature measurement during the tensile test. The Portevine–Le Chatelier effect (the so-called PLC effect) was observed for tests of specimens cut from sheet plates, which was strongly reflected in the temperature fluctuations. The course of temperature change during tensile tests was divided into four characteristic stages related to the occurrence of a clear or offset yield strength. It was found that if there is a clear yield strength, the value of the thermoelasto-plastic limit stress was greater than this yield strength. If there was an offset yield strength, the value of the thermoelasto-plastic limit stress was lower than this yield strength. The differences in the aforementioned values of individual yield strengths did not exceed several percent. Thus, it can be concluded that the thermoelasto-plastic limit stress determined on the basis of the course of specimen temperature changes during the tensile test is well correlated with the value of the yield strength of the material.

## 1. Background

Various strain processes occur during the loading of the structural member or a material specimen, depending on the load value, material type, and its microstructure and physical conditions of loading, such as the ambient temperature. Different mechanisms of microscopic material deformation, associated mainly with its dislocation structure, manifest in the macroscopically perceptible strain [1]. The relationship between mechanical properties and the material structure, particularly, dislocation as well as their effect on the material behavior in engineering structures is described in more detail, e.g., in the study [2]. This study focuses mainly on the macroscopic relationship between strain and stress and the associated change of the tested object temperature.

**Figure 1:**
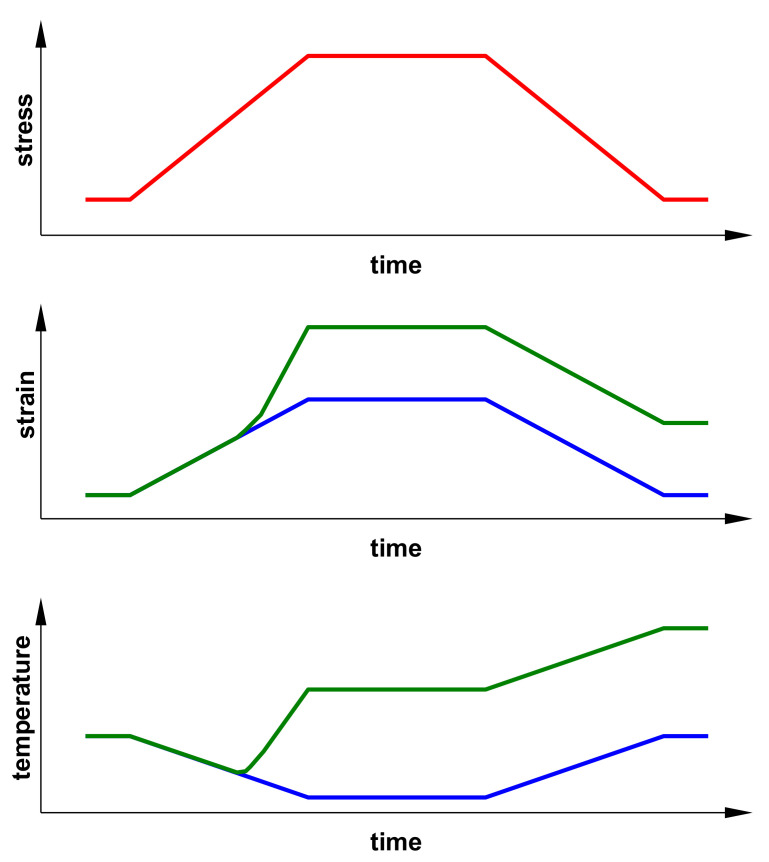
Schematic representation of the change in the course of strain and temperature over time due to trapezoidal load for a material in the elastic state (blue lines) and in an elastic-plastic state (green lines) (on the basis of [1]).

Figure 1 shows schematically the change in the course of strain and temperature over time during a trapezoidal load for a material in an elastic state and in an elastic-plastic state. A particularly important difference can be observed in the temperature course. An increase in load and strain in the case of material in an elastic state results in a temperature drop in line with the thermoelastic effect, while a drop in load increases the temperature up to the initial value.

In the thermoelastic effect, the elastic strain of the material causes material temperature change in adiabatic conditions. The temperature of the material under compressive stress increases, while under tensile stress (in certain circumstances) it decreases. During the adiabatic loading of metal by fast tension, its volume slightly changes. A small energy amount necessary to such a volume change may be provided at the expense of thermal energy, which causes a temperature drop. During slow, isothermal loading, the temperature evens out fast enough to make the thermoelastic effect unnoticeable. The thermoelastic effect consists of the reverse conversion between mechanical and thermal energy, with the accompanying elastic tension or compression causing temperature change. This effect is not the only underlying reason for the tested object temperature change—the temperature can change also due to external heat source, thermal conductivity, and internal energy dissipation.

First experiments on the thermoelastic effect are ascribed to Wilhelm Weber (1830) [3], although some mentions of that phenomenon can also be found in Gough’s papers (1805) [4]. The theoretical basis of thermoelastic effect were defined by William Thomson (later known as Lord Kelvin) in 1853 [5].

The phenomenon described above is used in Thermoelastic Stress Analysis (TSA) based on the linear relationship between the temperature change of a isotropic solid body and the change of total principal stresses [6].

(1) $$\Delta T=-\frac{\alpha }{\rho \cdot C}\cdot T_{0}\cdot \Delta S=-K_{m}\cdot T_{0}\cdot \Delta S,$$

where: *α*—linear thermal expansion coefficient, *ρ*—density, *C*—specific heat, *T*_0_–reference temperature in Kelvin degrees, *K_m_*—thermoelastic material constant, Δ*S*—change of the total principal or normal stresses [7].

(2) $$\Delta S=\Delta \left(S_{1}+S_{2}+S_{3}\right)=\Delta \left(S_{x}+S_{y}+S_{z}\right),$$

while for the plane stress condition (object surface) *S_z_* = 0.

Since the TSA analyses result in the total stress, it is necessary to separate them into individual components [8]. The scope of application of the classic TSA is limited to the applicability scope of Equation (1).

The temperature change curve is different for material in the elastic-plastic state. The temperature decreases with the load increase, similarly as for elastic material, and it reaches the minimum value roughly at the moment of macroscopic plastic strain occurrence. It is the transition point between the prevailing thermoelastic cooling and prevailing thermoplastic heating of the material. Bottani and Caglioti [9,10] proposed to define the stress corresponding to the minimum temperature as the thermoelasto-plastic limit stress and designate it with *σ_θ_*. In the available literature, however, attempts can be hard found to link the course of temperature changes and the value of this limit with the occurrence of a clear or offset yield strength of the material.

Experimental tests are currently commonly carried out using thermographic cameras to measure the temperature change accompanying the load. Infrared thermography allows quick surface temperature measurements both in the case of tensile (e.g., [11,12,13,14]) and fatigue tests (e.g., [15,16,17]), where infrared thermography has been used primarily for the accelerated estimation of fatigue limit values and fatigue curves (e.g., [18,19,20,21,22,23]). This method is used for the analysis of both variable uniaxial as well as multi-axial loads (e.g., [24,25]).

The observation of the macroscopic temperature change during the tensile is the underlying principle of, among other things, the fatigue limit estimation method called Static Thermographic Method, where the variation of the slope of the stress strain versus temperature curve identifies the transition zone between thermoelastic and thermoplastic behavior when the beginning of irreversible micro-plasticization occurs. This moment is considered by the authors as the value of the fatigue limit of the tested material [26,27,28,29,30]. This method has also been used for notched metal specimens [31] Another approach to investigate the fatigue limit during tensile static tests was proposed in [32] where the authors estimated the dissipation and the area where the first increase of energy occurs. The macro average stress value applied on the specimen, when the first increase of temperature occurs, corresponds to the conventional fatigue limit of material.

In this study, the author attempts to generalize the description of the course of specimen temperature changes during the tensile test and to connect the value of the thermoelasto-plastic limit stress with the value of a clear or proof strength (offset yield strength) [33,34] on the example of tests of aluminum alloys used in Poland for airplane structures.

## 2. Materials and Methods

**Figure 2:**
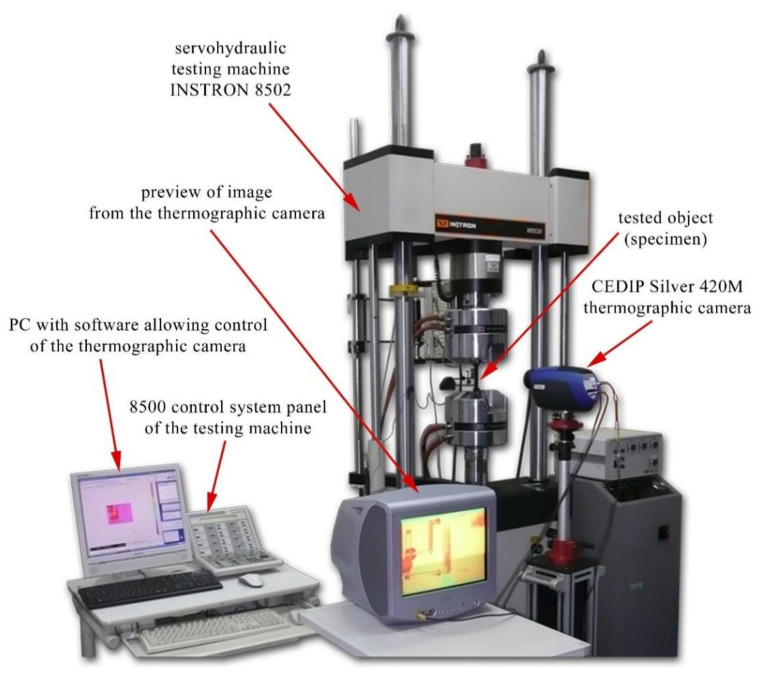
Test station with the thermographic measurement system.

The tests were performed in the Laboratory for Research on Materials and Structures of the Faculty of Mechanical Engineering at the UTP University of Science and Technology using the testing machine INSTRON 8502 presented in Figure 2 (with the following parameters: maximum static force: 300 kN, maximum dynamic force: 250 kN, piston stroke: ±75 mm) fitted with the control system 8500+. The laboratory is accredited by the Polish Centre for Accreditation (PCA AB 372) in compliance with ISO/IEC 17025:2018-02 and the accreditation covers, among others, testing methods used in this paper.

The main item of the test station was the thermographic camera CEDIP Silver 420M (FLIR SC5200) equipped with high sensitivity InSb matrix cooled using a Stirling pump. Main parameters of the camera: resolution of sensor 320 × 256 pixels, spectral range 3.6 ÷ 5.0 μm, sensitivity below 20 mK (available: 8 mK), maximum frequency 140 Hz for the entire matrix (up to 25 kHz at lower resolution). The thermographic camera recorded, at a constant frequency of 50 Hz, the surface temperature distribution of the tested object (specimen) fixed in the testing machine. Processing of thermographic images recorded by the camera was made by using ALTAIR and MATLAB software.

Monotonic tensile testing was carried out using specimens cut from sheet plates of different thicknesses made of typical aluminum alloys used in Poland for airplane structures: 2024-T3 alloy and its Russian equivalent, i.e., D16 alloy in the following three grades: D16CzATV, D16ATV, and D16UTV. Specimens were cut using Water-Jet technology (pre-treatment) in two mutually perpendicular directions, parallel to the sheet plate rolling direction and perpendicular to the sheet plate rolling direction, and then the side part, within the measuring range, remaining after the cutting operation, was subjected to the finishing treatment in order to obtain the required surface quality. The remaining surfaces of the specimens were left as delivered.

**Figure 3:**
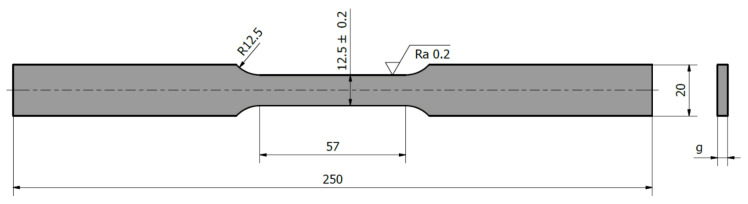
The geometry of test specimens (*g*-specimen thickness depending on the sheet plate thickness).

The tests were carried out at room temperature in accordance with the standard [33]. During tests, the specimens were subjected to uniaxial monotonically increasing tension up to their failure. The load force and the displacement of the test machine handle, together with the specimen strain, were recorded during tests using the INSTRON 2630-113 extensometer with the measurement base of 50 mm and the measuring range from −5 to +50 mm. The tests were carried out with displacement control at the speed of 0.05 mm/s. The geometry of the samples used is presented in Figure 3, with individual samples varying in thickness *g* depending on the thickness of the sheet plate. Five specimens were tested per each material grade and cutting direction.

The tests were carried out using specimens cut from 0.16” and 0.05” thick non-clad plates made of 2024-T3 aluminum alloy (AlCu4Mg1—supersaturated, cold deformed, and naturally aged up to stable condition) The chemical composition as regards the remaining components of 2024-T3 aluminum alloy is shown in Table 1.

The tests of D16 aluminum alloy were carried out using sheet plates of three grades differing additionally in thickness:

(a) D16ATV with a thickness of 1.13 mm,
(b) D16UTV with a thickness of 1.11 mm,
(c) D16CzATV with a thickness of 0.55 mm,

where the corresponding letters in the D16 alloy designation specify: V (or W)—plate used for airplane plating, A—clad plate with normal clad made of AD1 alloy, U—clad plate with thick clad, T—supersaturated and naturally aged metal plate, Cz—plate of increased purity in terms of chemical composition.

The chemical composition of the alloys tested is given in Table 2 and Table 3.

## 3. Results

### 3.1. Sheet Plates of 2024-T3 Aluminum Alloy

**Figure 4:**
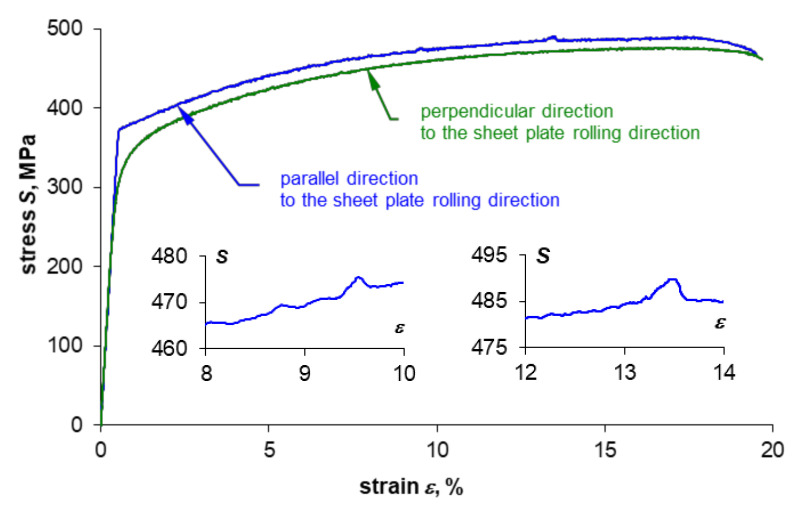
Tensile test curves for specimens made of 2024-T3 aluminum alloy in the form of 0.16” thick sheet plate.

**Figure 5:**
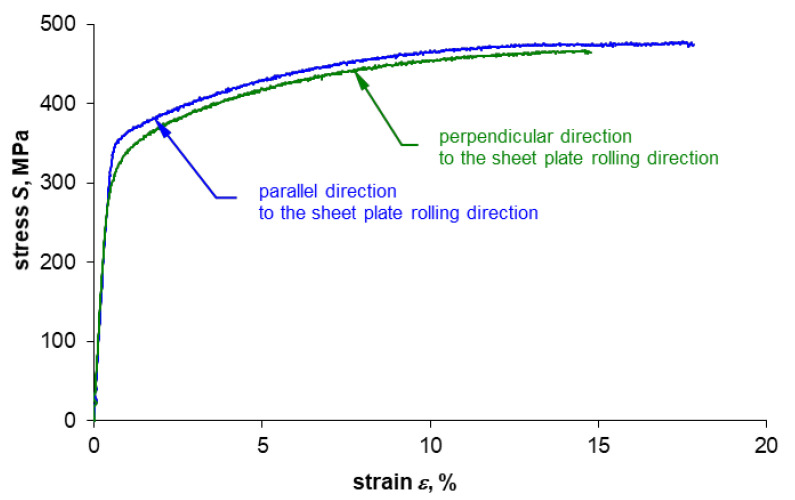
Tensile test curves for specimens made of 2024-T3 aluminum alloy in the form of 0.05” thick sheet plate.

Sample curves of tensile tests for both sampling directions are shown in Figure 4 and Figure 5. The monotonic properties obtained are provided in Table 4 and Table 5. Five samples were tested per each plate thickness and each sampling direction.

**Figure 6:**
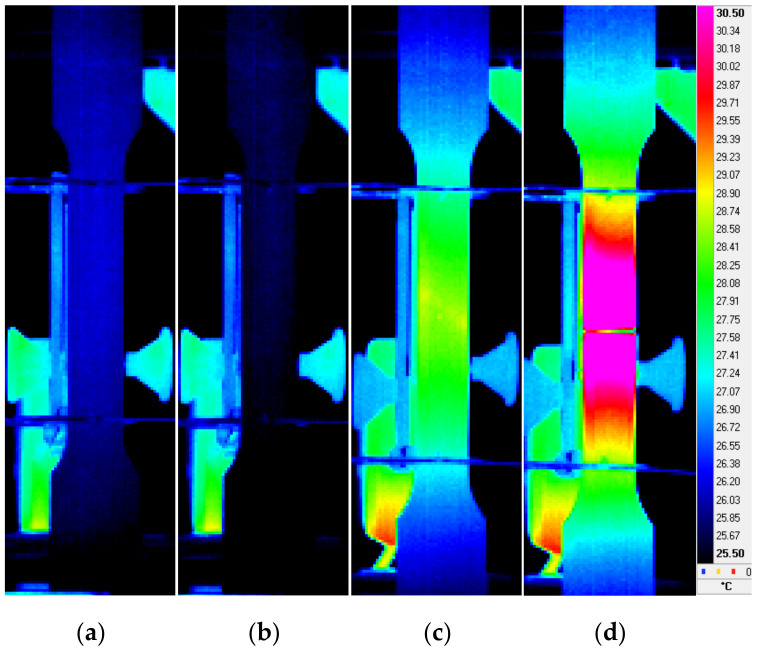
Selected specimen surface temperature distributions (**a**–**d**) obtained during the monotonic tensile test of 0.05” thick 2024-T3 aluminum alloy sheet plate.

Sample surface temperature distributions, typical for the selected characteristic stages of the monotonic tensile test are shown in Figure 6. The Figure 6a shows the temperature distribution corresponding to the beginning of the test (approx. T = 26.2 °C). As the specimen loading begins, its temperature starts to fall and reaches the minimum value (Figure 6b—approx. T = 25.7 °C)) and then increases until the moment just before the specimen failure (Figure 6c—approx. T = 28.5 °C). At the beginning of the failure (cracking) the specimen temperature reached the maximum value of approx. T = 52.3 °C. The Figure 6d shows the temperature distribution recorded 1 s after the beginning of failure (cracking) during the specimen cooling after the test completion.

### 3.2. Sheet Plates of D16 Aluminum Alloy

The monotonic properties obtained for D16 aluminum alloy sheet plates are provided in Table 6, Table 7 and Table 8. Five samples were tested per each sheet plate material grade and each sampling direction.

## 4. Discussion

### 4.1. Sheet Plates of 2024-T3 Aluminum Alloy

**Figure 7:**
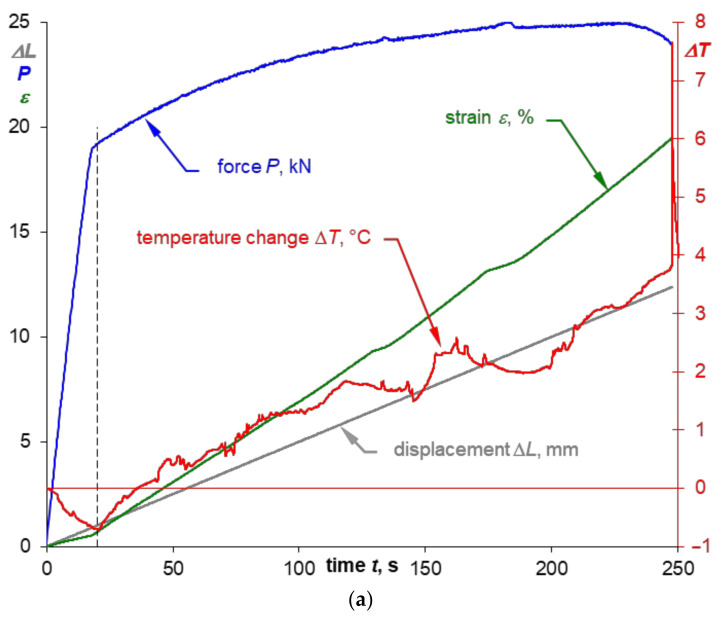

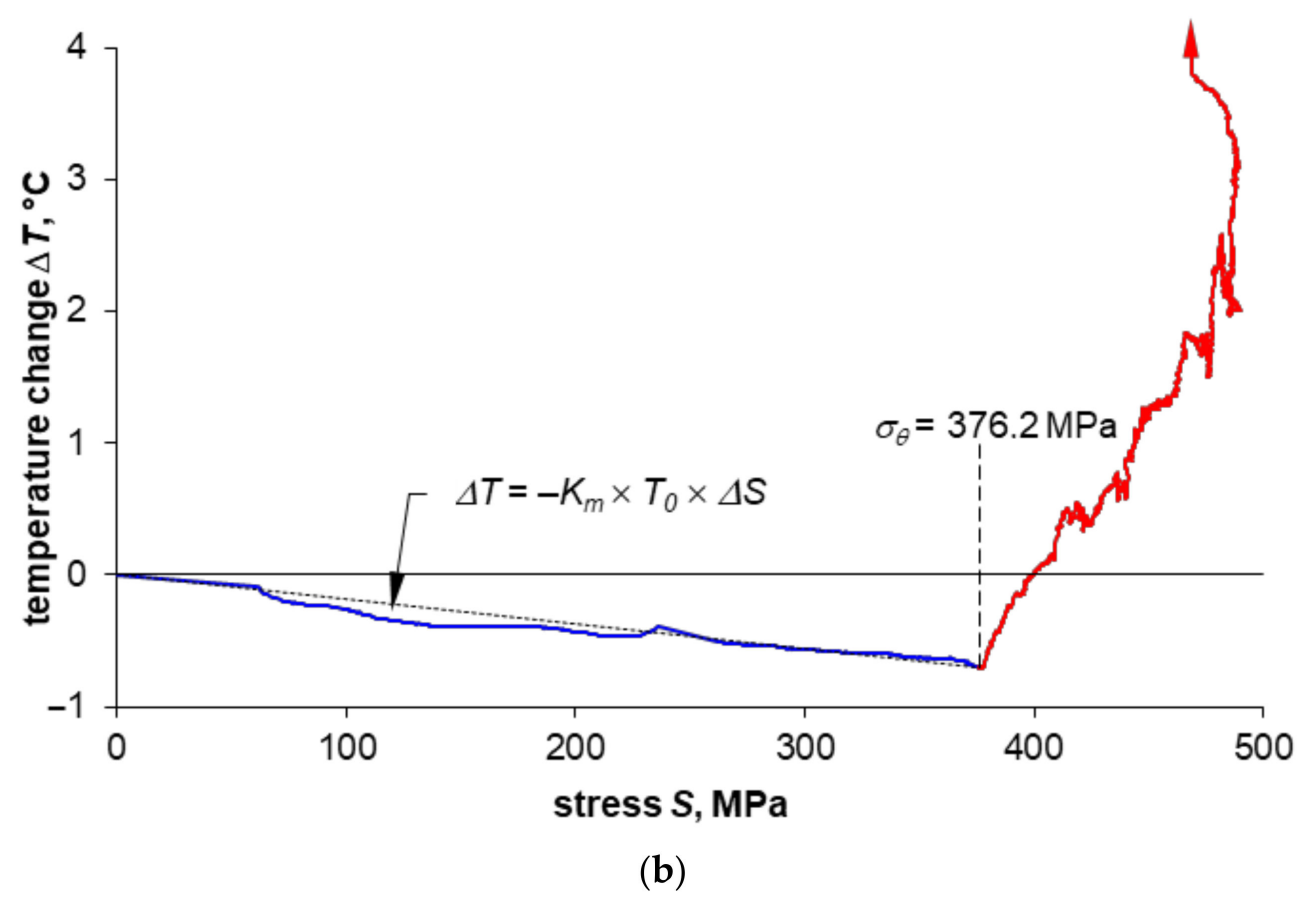
Sample time courses of the parameters recorded during the monotonic tensile test (**a**) for a specimen made of 0.16” thick 2024-T3 sheet plate cut parallel to the rolling direction and the temperature-strain relation (**b**) including the marking of the thermoelasto-plastic limit stress *σ_θ_*.

**Figure 8:**
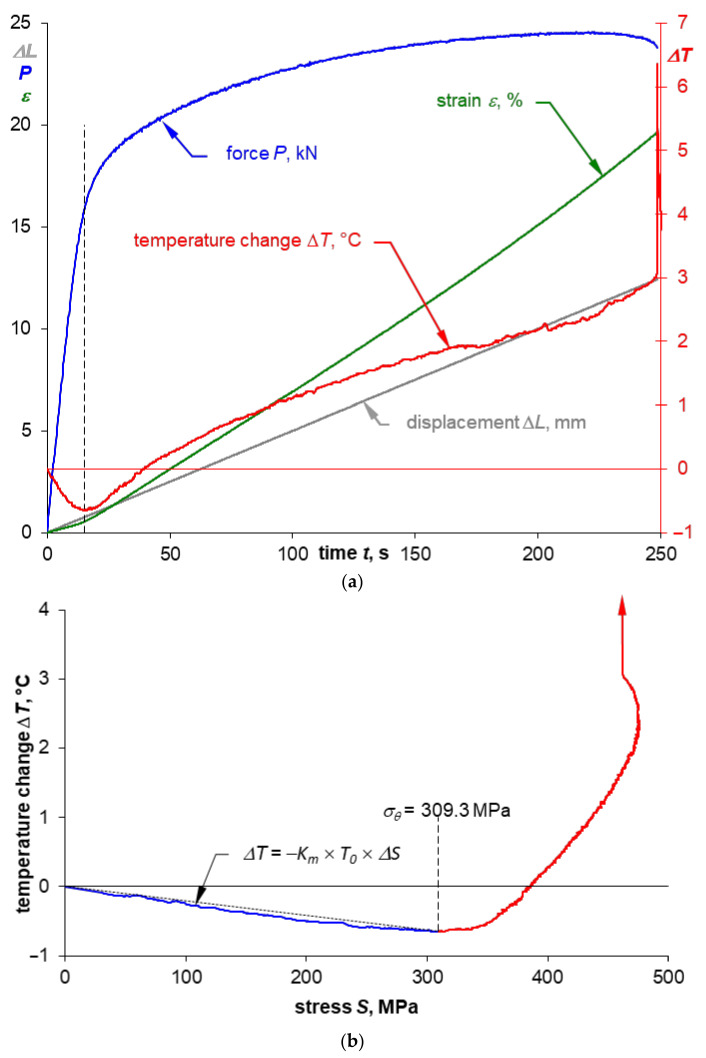
Sample time courses of the parameters recorded during the monotonic tensile test (**a**) for a specimen made of 0.16” thick 2024-T3 sheet plate cut perpendicular to the rolling direction and the temperature-strain relation (**b**) including the marking of the thermoelasto-plastic limit stress *σ_θ_*.

The summary of the values recorded during the test (force, strain, and displacement) together with the course of temperature change on the example of specimens made of 0.16” thick 2024-T3 sheet plates for both sampling directions are presented in Figure 7a and Figure 8a. Comparison of the presented courses shows, first of all, that the tested sheet plate demonstrates a clear yield strength in the sheet plate rolling direction, unlike in the direction perpendicular to the sheet plate rolling direction, where it does not show such yield strength. This fact is also reflected in the temperature course at the moment of specimens macroscopic plastic strain occurrence: there is a smooth transition through the minimum temperature (which is marked with a dashed line) for specimens with no clear yield strength and abrupt transition for samples characterized by a clear yield strength.

Comparison of the course of tensile curves for both sampling directions (Figure 4 and Figure 5) shows that the stress gradients are similar in the range of macroscopic plastic strain, but for the rolling direction one can notice momentary peaks of stress values, which are particularly high for the 0.16” thick sheet plate, as shown enlarged in Figure 4. However, the biggest differences depending on the sheet plate sampling direction can be observed in the course of temperature changes once the elastic-plastic point is exceeded. Significant temperature fluctuations (Figure 7a) are visible for the specimens cut parallel to the rolling direction, which can be related to the occurrence of stress peaks. The temperature curve for the specimens cut perpendicular to the rolling direction is much more stable (Figure 8a).

The abrupt stress change during the tensile test shown above, observed for the specimens cut parallel to the rolling direction, is characteristic of the Portevin–Le Chatelier effect (the so-called PLC effect) occurring, among others, for non-ferrous metal alloys (e.g., brass and aluminum alloys) as well as for iron at elevated temperatures.

The Portevin–Le Chatelier effect manifests in the tensile curve with multiple repeating peaks which result from the fact that atoms of impurities are captured by moving dislocations, which are immobilized and then suddenly released. Blocking dislocation movement results in stress increase, while the release of dislocation results in stress reduction. As a result, the dislocation movement rate changes between extreme values. At low velocities, the strain progress caused by stress increase is slow and once it achieves sufficient value for dislocation release, the fast plastic flow phase starts with a decrease in stress. Released dislocations capture atoms of impurities on their way again, which results in dislocation slowing down, and that cycle repeats again. It should be noted that the PLC effect takes place only within a certain limited strain rate range. For a sufficiently high strain rate, the flow stress is always higher than the dislocation release stress and, as a result, an abrupt strain change is not observed [2].

**Figure 9:**
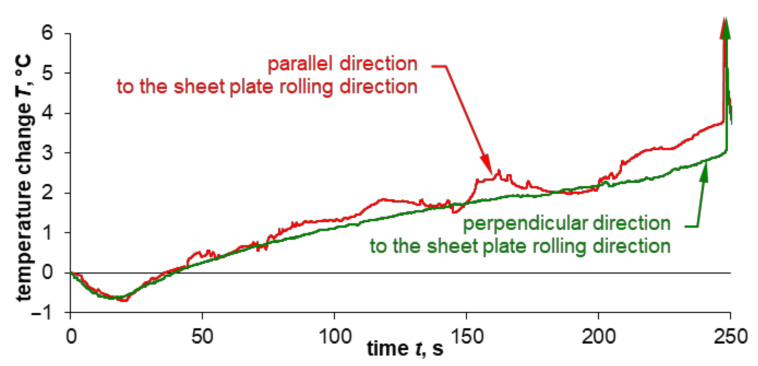
The summary of the temperature change course for specimens cut out of 0.16” thick 2024-T3 sheet plate parallel and perpendicular to the rolling direction.

The Figure 9 provides a summary of the courses of temperature change for specimens depending on their cutting direction from the sheet plate. The curve obtained for the sampling direction perpendicular to the rolling direction, for which no Portevin–Le Chatelier effect was found, represents the lower limit for the curve, for which this effect is clearly visible. It may therefore be concluded that the PLC effect is responsible for the recorded temperature fluctuations for the specimens cut in-line with the rolling direction, and for its momentary increases from a value approximately corresponding to the one observed for specimens cut perpendicular to the rolling direction.

Figure 7b and Figure 8b show selected stress-temperature curves obtained on the basis of the time courses presented in Figure 7a and Figure 8a. On these diagrams the minimum value of temperature was marked with the corresponding value of the thermoelasto-plastic limit stress *σ_θ_*. A summary of the yield strength and the thermoelasto-plastic limit stress values for sheet plates made of the 2024-T3 alloy is presented in Table 9. The comparison shows that in the case of the material demonstrating a clear yield strength, the thermoelasto-plastic limit stress is higher than the yield strength (by 4.1% maximum for tests of 2024-T3 sheet plates). This may be related to the fact that the thermoelastic cooling effect prevails up to the value of the yield strength, while once the yield strength is exceeded, the thermoplastic heating of the material becomes more and more significant. Those two effects are balanced at the point corresponding to the thermoelasto-plastic limit stress, i.e., the heating balances the cooling that resulted from the thermoelastic effect. In the case of a material for which an offset yield strength is determined, the thermoelasto-plastic limit stress is lower than the offset yield strength (by 5.7% maximum for the performed tests of 2024-T3 sheet plate specimens). In this case, the plastic strain of a value sufficient to balance the thermoelastic cooling occurs much earlier than in the results from the assumed value of the offset yield strength. This conclusion is in line with the observations presented in the paper [1] concerning research on austenitic steel with no clear yield strength, where the difference reached 10% and decreased with decrease in the gradient of the transition from elastic to plastic stress.

A line corresponding to the range for which TSA (Thermoelastic Stress Analysis) is possible is also marked in the Figure 7b and Figure 8b. A summary of the average values for the thermoelastic material constant *K_m_* [37] obtained from the test results for 2024-T3 sheet plate is given in Table 10. The value of the material constant *K_m_* depends on the sheet plate rolling direction—i.e., it is much higher when perpendicular to the rolling direction. Smaller differences are observed depending on the thickness—i.e., a thicker sheet plate shows slightly higher *K_m_* value than thinner one.

**Figure 10:**
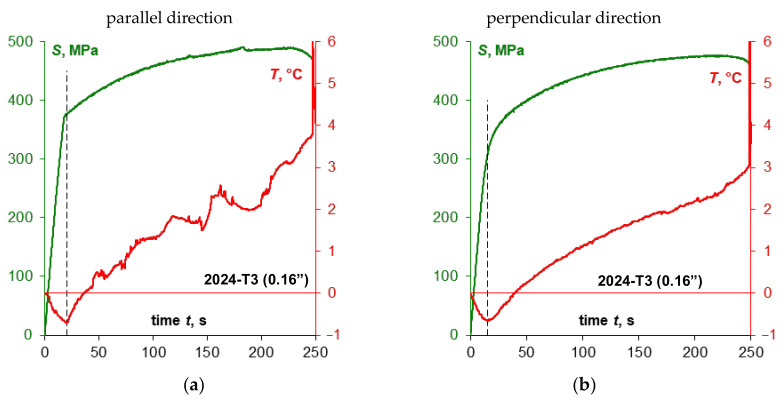

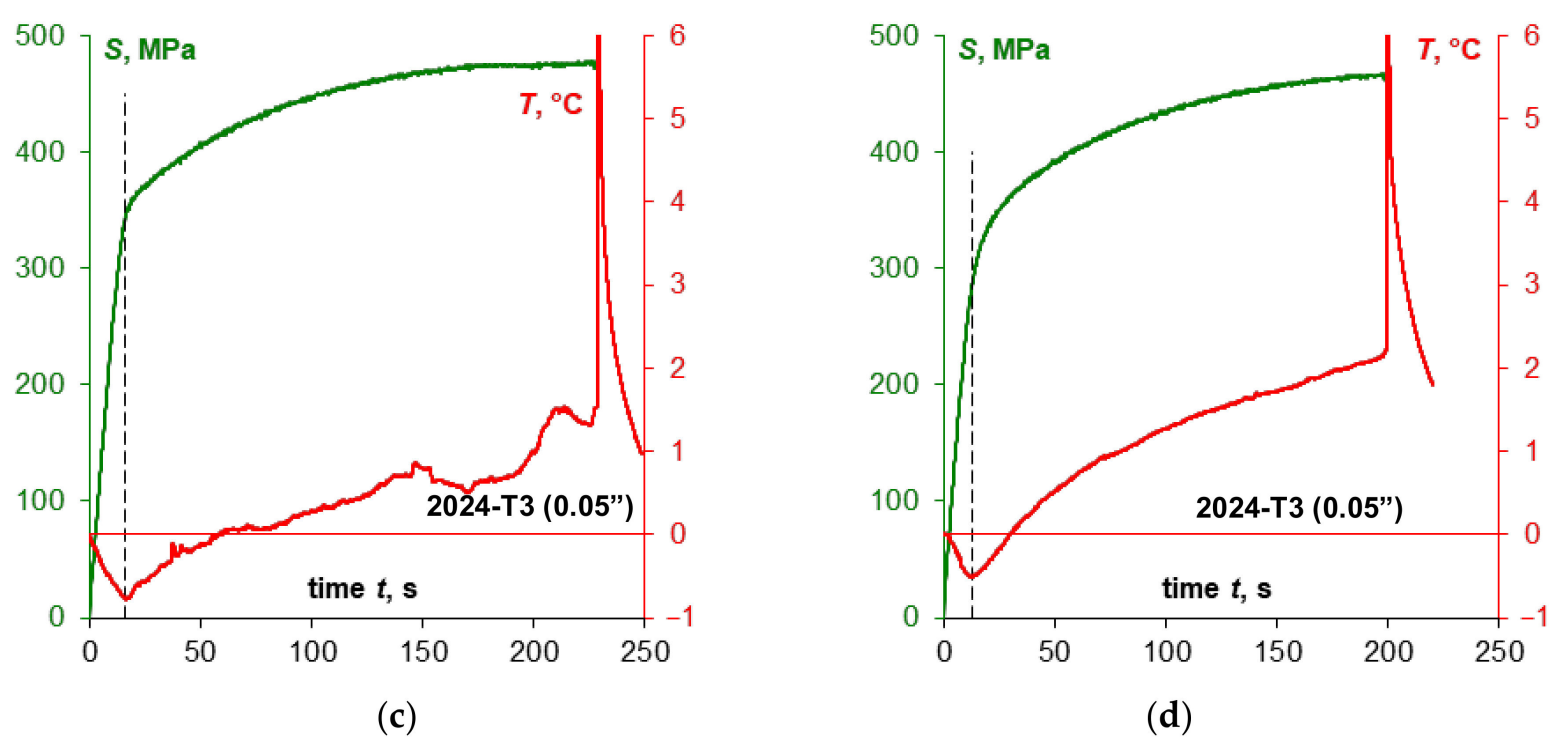
The summary of stress *S* and temperature *T* change curves obtained during monotonic tensile tests of specimens made of 0.16” thick 2024-T3 sheet plate (**a**,**b**) and 0.05” thick 2024-T3 sheet plate (**c**,**d**) depending on the specimen cutting direction in relation to the sheet plate rolling direction.

**Figure 11:**
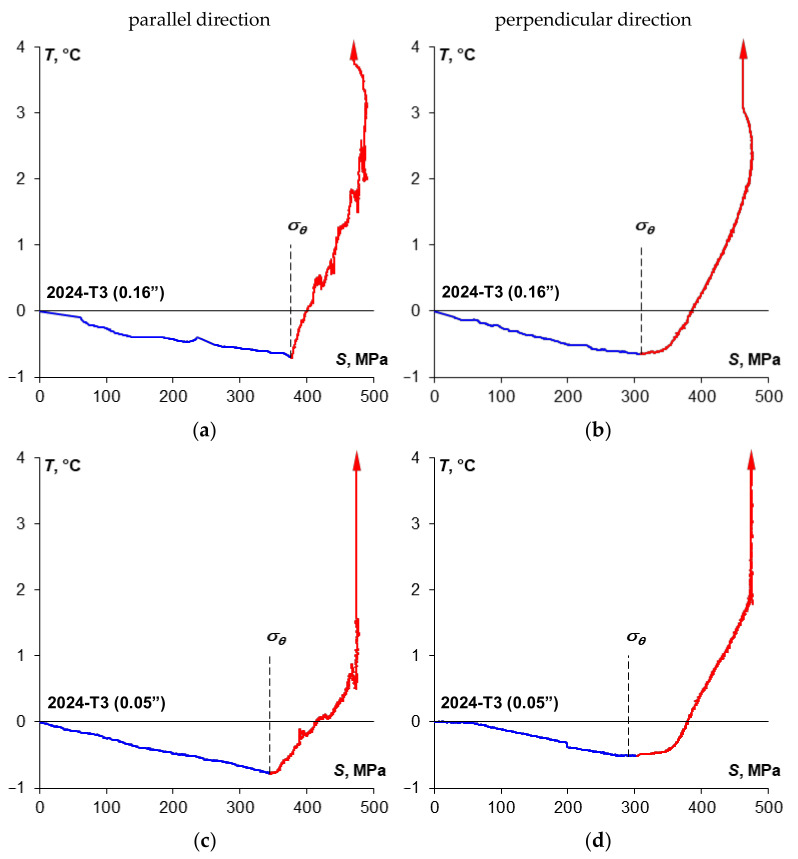
The summary of the temperature *T* and stress *S* change curves obtained during monotonic tensile tests of specimens made of 0.16″ thick 2024-T3 sheet plate (**a**,**b**) and 0.05” thick 2024-T3 sheet plate (**c**,**d**) depending on the specimen cutting direction in relation to the sheet plate rolling direction.

As a summary of the thermographic description of the monotonic tensile test of specimens made of 2024-T3 aluminum alloy sheet plate, the time curves of stress *S* and temperature change *T* are presented in Figure 10, while Figure 11 shows time curves of temperature change *T* versus stress *S* depending on the thickness and the sample cutting direction in relation to the sheet plate rolling direction. This summary confirms the observations described above for the analyzed material.

### 4.2. Sheet Plates of D16 Aluminum Alloy

**Figure 12:**
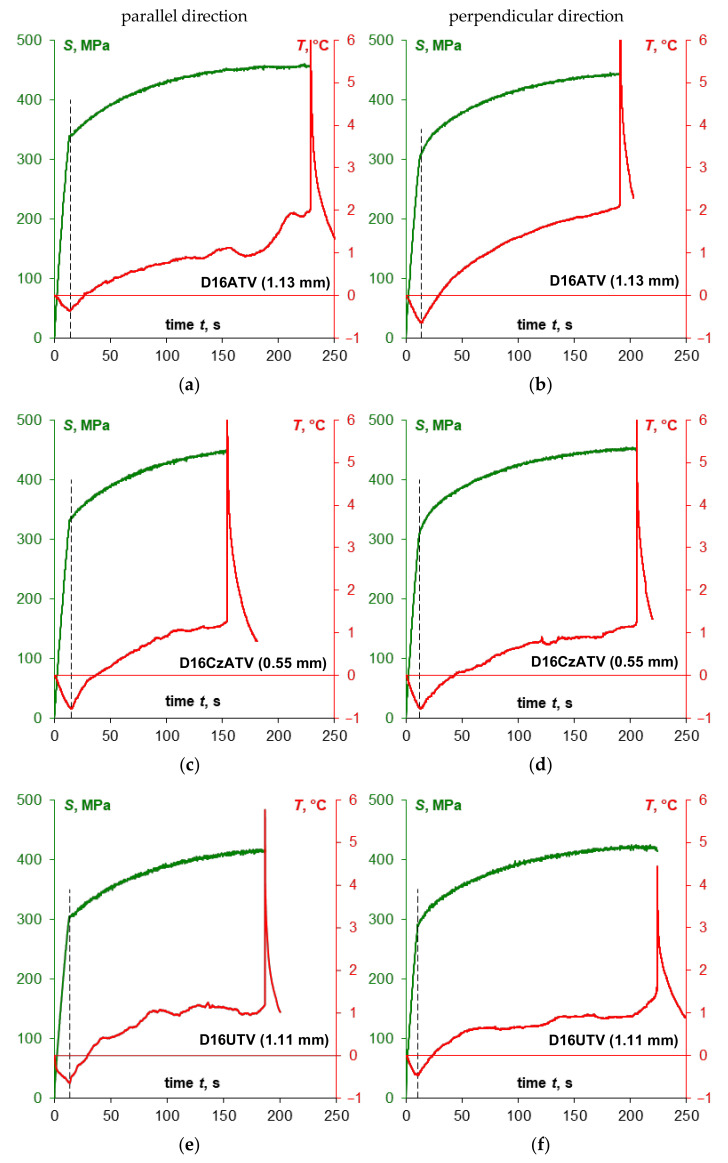
The summary of stress *S* and temperature *T* change curves obtained during monotonic tensile tests of specimens made of D16ATV sheet plate (**a**,**b**), D16CzATV sheet plate (**c**,**d**) and D16UTV sheet plate (**e**,**f**) depending on the specimen cutting direction in relation to the sheet plate rolling direction.

**Figure 13:**
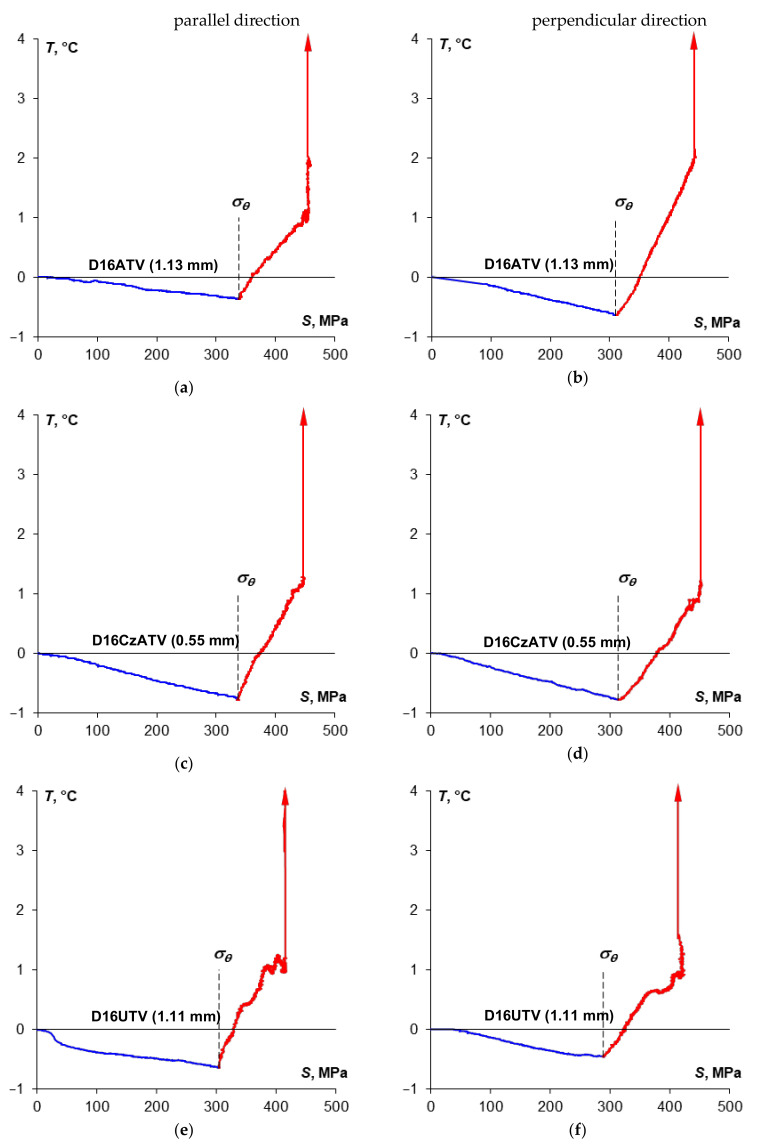
The summary of the temperature *T* and stress S change curves obtained during monotonic tensile tests of specimens made of D16ATV sheet plate (**a**,**b**), D16CzATV sheet plate (**c**,**d**) and D16UTV sheet plate (**e**,**f**) depending on the specimen cutting direction in relation to the sheet plate rolling direction.

The Figure 12 shows the summary of stress *S* and temperature *T* change curves, while the Figure 13 shows time curves of temperature change *T* vs. stress *S* obtained during monotonic tensile tests of specimens made of D16 sheet plate depending on the specimen cutting direction in relation to the sheet plate rolling direction.

In the case of D16ATV and D16UTV sheet plates, there is a clear yield strength in both sampling directions, which is also reflected in the specimen temperature curve, where a sharp transition for both directions has been found in the thermoelasto-plastic limit stress area. For D16CzATV sheet plate, like in the case of 2024-T3, there is a clear yield strength and a sharp transition in the thermoelasto-plastic limit stress area visible in Figure 13c, while a smooth transition (Figure 13d) is associated with the lack of a clear yield strength.

The Portevin–Le Chatelier effect was always noticeable in the direction parallel to the sheet plate rolling direction and was strongly marked for the D16ATV alloy. For D16CzATV and D16UTV alloys the PLC effect was also present for the direction perpendicular to the rolling direction, with temperature fluctuations lower than in the parallel direction.

The Table 11 includes the summary of the yield strength values and thermoelasto-plastic limit stress values. Like in the case of the 2024-T3 alloy, if there is a clear yield strength, the value of the thermoelasto-plastic limit stress was higher than the yield strength (by 9.9% maximum for D16UTV), while lower compared to the offset yield strength (by 5.3% maximum for D16CzATV). In case of D16ATV alloy, the values of the yield strength and the thermoelasto-plastic limit stress were close to each another.

The Table 12 provides the summary of the experimentally determined thermoelastic constant values. The values obtained are close to each other for the specimens cut perpendicular to the sheet plate rolling direction. Significant differences are observed for individual materials depending on the sheet plate rolling direction.

## 5. Conclusions

**Figure 14:**
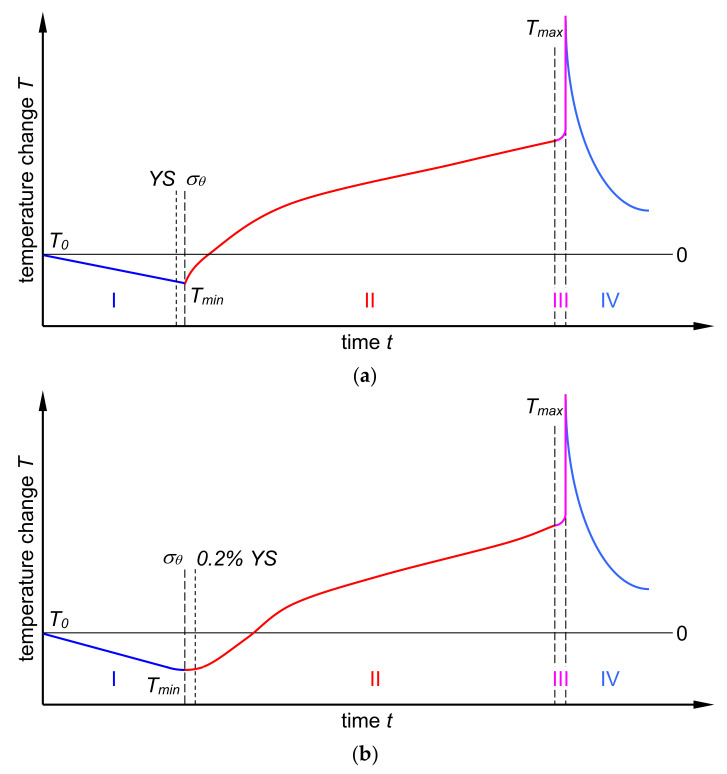
Schematic representation of the course of temperature change during the monotonic tension test of the material featuring a clear yield strength (**a**) and no clear yield strength (**b**).

On the basis of the above examples of monotonic tensile of specimens made of airplane aluminum alloy sheets, the temperature change during loading can be divided into several phases (Figure 14):

**Phase I**. The phase corresponding to the elastic strain of the specimen for which there is a linear relationship between the temperature change and the stress value, and which can be described using relationship (1).

In the case of materials featuring a clear yield strength (Figure 14a), this phase ends with a sudden transition between the prevailing thermoelastic cooling range and the thermoplastic heating range corresponding to the thermoelasto-plastic limit stress *σ_θ_*. The value of the thermoelasto-plastic limit stress *σ_θ_* is higher than the value of the clear yield strength YS.

In the case of materials that do not feature a clear yield strength (Figure 14b), this phase ends with a smooth transition between the predominant thermoelastic cooling range and the thermoplastic heating range. The value of the thermoelasto-plastic limit stress *σ_θ_* is lower than the value of the offset yield strength 0.2% YS.

**Phase II**. The phase corresponding to the plastic strain of the specimen, while the higher stress gradient is associated with higher temperature gradient.

In the case of materials featuring a clear yield strength, this phase starts with a steep rise in temperature.

In the case of materials that do not feature a clear yield strength, this phase starts with a smooth transition from phase I associated with the nature of the stress transition from elastic to plastic one.

**Phase III**. Phase of the macroscopic crack development in the specimen. It ends with a steep temperature rise up to the maximum value corresponding to total failure of the specimen. This phase is very short as compared to the other phases.

**Phase IV**. Phase of the specimen cooling down to the ambient temperature after total failure of the specimen due to fracture.

## Figures and Tables

**Table 1:** The content of main components (apart from aluminum) of 2024-T3 alloy (wt %).

		Si	Fe	Cu	Mn	Mg	Cr	Zn	Ti
according to standard [35]	min	-	-	3.8%	0.3%	1.2%	-	-	-
	max	0.50%	0.50%	4.9%	0.9%	1.8%	0.1%	0.25%	0.15%
thickness 0.16”		0.07%	0.16%	4.7%	0.65%	1.5%	0.00%	0.10%	0.03%
thickness 0.05”		0.06%	0.16%	4.7%	0.63%	1.5%	0.01%	0.16%	0.03%

**Table 2:** The content of main chemical components (apart from aluminum) of D16ATV and D16UTV alloys (wt %).

		Si	Fe	Cu	Mn	Mg	Cr	Zn	Ti
according to standard [36]	min	-	-	3.8%	0.3%	1.2%	-	-	-
	max	0.50%	0.50%	4.9%	0.9%	1.8%	0.1%	0.25%	0.15%
D16ATV		0.23%	0.26%	3.88%	0.38%	1.38%	0.01%	0.01%	0.03%
D16UTV		0.25%	0.26%	3.93%	0.43%	1.41%	0.01%	0.01%	0.03%

**Table 3:** Chemical composition of main components (apart from aluminum) of D16CzATV alloy(wt %).

		Si	Fe	Cu	Mn	Mg	Cr	Zn	Ti
according to standard [36]	min	-	-	3.8%	0.3%	1.2%	-	-	-
	max	0.20%	0.30%	4.9%	0.9%	1.8%	0.1%	0.25%	0.15%
D16CzATV		0.11%	0.22%	3.91%	0.34%	1.33%	0.03%	0.01%	0.02%

**Table 4:** Monotonic properties of the 2024-T3 alloy in the form of 0.16” thick sheet plate.

Direction of Cut Out Specimens from a Sheet Plate	Young’s Modulus	Yield Strength	0.2% Yield Strength	Ultimate Tensile Strength	Elongation
	GPa	MPa	MPa	MPa	%
parallel to the rolling direction	67.6 ± 3.3	362.3 ± 2.0	-	488.3 ± 1.0	19.4 ± 0.4
perpendicular to the rolling direction	69.7 ± 5.0	-	324.2 ± 1.3	479.0 ± 2.0	20.3 ± 0.9

**Table 5:** Monotonic properties of the 2024-T3 alloy in the form of 0.05” thick sheet plate.

Direction of Cut Out Specimens from a Sheet Plate	Young’s Modulus	Yield Strength	0.2% Yield Strength	Ultimate Tensile Strength	Elongation
	GPa	MPa	MPa	MPa	%
parallel to the rolling direction	68.9 ± 2.3	334.0 ± 3.4	-	468.7 ± 3.5	15.9 ± 1.5
perpendicular to the rolling direction	70.2 ± 1.4	-	311.5 ± 3.4	461.0 ± 5.6	15.1 ± 0.9

**Table 6:** Monotonic properties of the D16ATV aluminum alloy in the form of 1.13 mm thick sheet plate.

Direction of Cut Out Specimens from a Sheet Plate	Young’s Modulus	Yield Strength	0.2% Yield Strength	Ultimate Tensile Strength	Elongation
	GPa	MPa	MPa	MPa	%
parallel to the rolling direction	69.8 ± 3.5	339.5 ± 4.8	-	463.9 ± 2.7	18.5 ± 1.5
perpendicular to the rolling direction	68.1 ± 0.2	303.0 ± 14.4	-	443.4 ± 12.9	15.0 ± 0.8

**Table 7:** Monotonic properties of the D16CzATV aluminum alloy in the form of 1.11 mm thick sheet plate.

Direction of Cut Out Specimens from a Sheet Plate	Young’s Modulus	Yield Strength	0.2% Yield Strength	Ultimate Tensile Strength	Elongation
	GPa	MPa	MPa	MPa	%
parallel to the rolling direction	66.1 ± 1.3	332.1 ± 1.4	-	447.6 ± 1.1	12.5 ± 0.4
perpendicular to the rolling direction	69.3 ± 3.0	-	319.0 ± 7.4	444.4 ± 5.8	13.2 ± 0.7

**Table 8:** Monotonic properties of the D16UTV aluminum alloy in the form of 0.55 mm thick sheet plate.

Direction of Cut Out Specimens from a Sheet Plate	Young’s Modulus	Yield Strength	0.2% Yield Strength	Ultimate Tensile Strength	Elongation
	GPa	MPa	MPa	MPa	%
parallel to the rolling direction	58.0 ± 1.1	288.8 ± 3.6	-	404.1 ± 4.9	14.4 ± 0.3
perpendicular to the rolling direction	63.4 ± 1.7	266.6 ± 4.5	-	406.6 ± 9.4	17.0 ± 0.9

**Table 9:** A summary of the yield strength and the thermoelasto-plastic limit stress values for sheet plates made of the 2024-T3 aluminum alloy depending on the thickness and the rolling direction.

Parameter MPa	Sheet Plate Thickness			
	0.16”		0.05”	
	Sheet Plate Specimen Cutting with Regard to the Rolling Direction			
	Parallel	Perpendicular	Parallel	Perpendicular
Yield strength	362.3 ± 2.0	-	334.0 ± 3.4	-
0.2% yield strength	-	324.2 ± 1.3	-	311.5 ± 3.4
Thermoelasto-plastic limit stress *σ_θ_*	373.6 ± 2.2	316.0 ± 5.7	339.1 ± 5.2	295.2 ± 7.4

**Table 10:** A summary of the thermoelastic material constant *K_m_* for sheet plates made of the 2024-T3 aluminum alloy depending on the thickness and the rolling direction.

Thermoelastic Constant MPa^−1^	Sheet Plate Thickness			
	0.16”		0.05”	
	Sheet Plate Specimen Cutting with Regard to the Rolling Direction			
	Parallel	Perpendicular	Parallel	Perpendicular
*K_m_*	6.38 × 10^−6^	7.40 × 10^−6^	6.05 × 10^−6^	7.03 × 10^−6^

**Table 11:** A summary of the yield strength and the thermoelasto-plastic limit stress values for sheet plate made of the D16 aluminum alloy depending on the thickness and the rolling direction.

Parameter MPa	D16ATV		D16CzATV		D16UTV	
	Sheet Plate Specimen Cutting with Regard to the Rolling Direction					
	Parallel	Perpendicular	Parallel	Perpendicular	Parallel	Perpendicular
Yield strength	339.5 ± 4.8	303.0 ± 14.4	332.1 ± 1.4	-	266.6 ± 4.5	288.8 ± 3.6
0.2% yield strength	-	-	-	319.0 ± 7.4	-	-
Thermoelasto-plastic limit stress *σ_θ_*	338.1 ± 4.2	304.0 ± 8.5	338.1 ± 2.2	312.0 ± 1.7	289.1 ± 1.7	306.0 ± 2.2

**Table 12:** A summary of the thermoelastic material constant *K_m_* for sheet plate made of D16 aluminum alloy depending on the thickness and the rolling direction.

Thermoelastic Constant MPa^−1^	D16ATV		D16CzATV		D16UTV	
	Sheet Plate Specimen Cutting with Regard to the Rolling Direction					
	Parallel	Perpendicular	Parallel	Perpendicular	Parallel	Perpendicular
*K_m_*	3.79 × 10^−6^	6.92 × 10^−6^	7.66 × 10^−6^	6.57 × 10^−6^	4.41 × 10^−6^	6.26 × 10^−6^

## Data Availability

The data presented in this study are available on request from the corresponding author after obtaining permission of authorized person. The data are not publicly available due to data confidentiality resulting from the requirements of the accreditation.

## References

[1] Harwood N., Cummings W.M. (1991). Thermoelastic Stress Analysis.

[2] Courtney T.H. (2000). Mechanical Behavior of Materials.

[3] Weber W. (1830). Über die specifische Warme fester Korper insbesondere der Metalle. Ann. Physik Chemie.

[4] Gough J. (1805). A description of a property of Caoutchouc, or India rubber; with some reflections on the cause of the elasticity of this substance. Mem. Lit. Philos. Soc. Manch..

[5] Thomson W. (1853). On the Dynamical Theory of Heat, with numerical results deduced from Mr Joule’s equivalent of a Thermal Unit, and M. Regnault’s Observations on Steam. T. R. Soc. Edinb..

[6] Lee H.-T., Chen J.-C. (1991). Temperature effect induced by uniaxial tensile loading. J. Mater. Sci..

[7] Kobayashi A.S. (1993). Handbook on Experimental Mechanics.

[8] Inoue H., Hirokawa Y., Kishimoto K. (2004). Stress Separation in Thermoelastic Stress Analysis Using Nonlinearity of the Thermoelastic Effect. JSME Int. J. Ser. A Solid Mech. Mater. Eng..

[9] Bottani C.E., Caglioti G. (1982). Thermoelastic Instabilities in Metals. Phys. Scr..

[10] Bottani C.E., Caglioti G. (1982). Thermal emission: A probe to identify the critical point of the elastoplastic transition. Mater. Lett..

[11] Kumar J., Baby S., Kumar V. (2008). Thermographic studies on IMI-834 titanium alloy during tensile loading. Mater. Sci. Eng. A.

[12] Oliferuk W., Maj M., Litwinko R., Urbański L. (2012). Thermomechanical coupling in the elastic regime and elasto-plastic transition during tension of austenitic steel, titanium and aluminium alloy at strain rates from 10^−4^ to 10^−1^ s^−1^. Eur. J. Mech. A/Solids.

[13] Lipski A. (2014). Impact of the Strain Rate during Tension Test on 46Cr1 Steel Temperature Change. KEM.

[14] Lipski A., Boroński D. (2012). Use of Thermography for the Analysis of Strength Properties of Mini-Specimens. MSF.

[15] Berthel B., Chrysochoos A., Wattrisse B., Galtier A. (2008). Infrared Image Processing for the Calorimetric Analysis of Fatigue Phenomena. Exp. Mech..

[16] Audenino A.L., Crupi V., Zanetti E.M. (2003). Correlation between thermography and internal damping in metals. Int. J. Fatigue.

[17] De Finis R., Palumbo D., da Silva M.M., Galietti U. (2018). Is the temperature plateau of a self-heating test a robust parameter to investigate the fatigue limit of steels with thermography?. Fatigue Fract. Eng. Mater. Struct..

[18] Rosa G.L., Risitano A. (2000). Thermographic methodology for rapid determination of the fatigue limit of materials and mechanical components. Int. J. Fatigue.

[19] Amiri M., Khonsari M.M. (2010). Rapid determination of fatigue failure based on temperature evolution: Fully reversed bending load. Int. J. Fatigue.

[20] Cura F., Curti G., Sesana R. (2005). A new iteration method for the thermographic determination of fatigue limit in steels. Int. J. Fatigue.

[21] Lipski A. (2015). Thermographic Method Based Accelerated Fatigue Limit Calculation for Steel X5CrNi18-10 Subjected to Rotating Bending. Pol. Marit. Res..

[22] Lipski A. (2016). Rapid Determination of the S–N Curve for Steel by means of the Thermographic Method. Adv. Mater. Sci. Eng..

[23] Lipski A. (2016). Accelerated Determination of Fatigue Limit and S-N Curve by Means of Thermographic Method for X5CrNi18-10 Steel. Acta Mech. Autom..

[24] Doudard C., Poncelet M., Calloch S., Boue C., Hild F., Galtier A. (2007). Determination of an HCF criterion by thermal measurements under biaxial cyclic loading. Int. J. Fatigue.

[25] Skibicki D., Lipski A., Pejkowski Ł. (2018). Evaluation of plastic strain work and multiaxial fatigue life in CuZn37 alloy by means of thermography method and energy-based approaches of Ellyin and Garud. Fatigue Fract. Eng. Mater. Struct..

[26] Clienti C., Fargione G., La Rosa G., Risitano A., Risitano G. (2010). A first approach to the analysis of fatigue parameters by thermal variations in static tests on plastics. Eng. Fract. Mech..

[27] Risitano A., Risitano G. (2013). Determining fatigue limits with thermal analysis of static traction tests. Fatigue Fract. Eng. Mater. Struct..

[28] Risitano A., Giacomo R., Clienti C. (2010). Fatigue limit by thermal analysis of specimen surface in mono axial traction test. EPJ Web Conf..

[29] Corigliano P., Cucinotta F., Guglielmino E., Risitano G., Santonocito D. (2019). Thermographic analysis during tensile tests and fatigue assessment of S355 steel. Procedia Struct. Integr..

[30] Corigliano P., Cucinotta F., Guglielmino E., Risitano G., Santonocito D. (2019). Fatigue assessment of a marine structural steel and comparison with Thermographic Method and Static Thermographic Method. Fatigue Fract. Eng. Mater. Struct..

[31] Foti P., Santonocito D., Ferro P., Risitano G., Berto F. (2020). Determination of Fatigue Limit by Static Thermographic Method and Classic Thermographic Method on Notched Specimens. Procedia Struct. Integr..

[32] Risitano A., Corallo D., Guglielmino E., Risitano G., Scappaticci L. (2017). Fatigue assessment by energy approach during tensile tests on AISI 304 steel. Frat. Integr. Strut..

[33] (2019). Standard EN ISO 6892-1:2019 Metallic Materials—Tensile Testing—Part 1: Method of Test at Room Temperature.

[34] (2016). Standard ASTM E8/E8M-16a Standard Test Methods for Tension Testing of Metallic Materials.

[35] (1997). Standard AMS QQA 250/4 Aluminum Alloy 2024, Plate and Sheet.

[36] (1998). Standard GOST 4784-97 Wrought Aluminium and Aluminium Alloys Grades.

[37] Sharpe W.N. (2008). Springer Handbook of Experimental Solid Mechanics.

